# Depolymerization and Oxidation Events in Used Frying Oils Under Conditions Simulating Gastric Digestion

**DOI:** 10.3390/foods14060925

**Published:** 2025-03-08

**Authors:** Gloria Márquez-Ruiz, María Victoria Ruiz-Méndez, Francisca Holgado

**Affiliations:** 1 Instituto de Ciencia y Tecnología de los Alimentos y Nutrición (ICTAN), Consejo Superior de Investigaciones Científicas (CSIC), José Antonio Novais, 10, 28040 Madrid, Spain; f.holgado@ictan.csic.es; 2 Instituto de la Grasa (IG), Consejo Superior de Investigaciones Científicas (CSIC), Campus/Bd 46, Ctra. de Utrera km 1, 41013 Sevilla, Spain; mvruiz@ig.csic.es

**Keywords:** depolymerization, lipid oxidation, frying, polymers, in vitro gastric digestion

## Abstract

The chemical modifications occurring to the multitude of compounds formed in oils during frying after ingestion and prior to absorption are still unknown. The objective of this work was to explore the depolymerization and oxidation events which may occur under simulated gastric conditions and obtain quantitative data of the compounds formed. Samples of used frying sunflower oil with increasing alteration degree were selected for in vitro digestion. The methodology applied to determine changes in triacylglycerols (TAG), oxidized TAG monomers (oxTAGM), TAG dimers (TAGD) and higher oligomers (TAGO) consisted of a combination of adsorption and size exclusion chromatographies while changes in epoxy, hydroxy and keto fatty acyls were evaluated after oil transesterification by combination of adsorption and gas–liquid chromatographies. Among the results obtained, the large extent of depolymerization after digestion at pH 1.2, reaching levels as high as 70%, stood out. The release of unoxidized TAG from polymeric molecules was reflected in their significant increase after digestion. Hydroxy fatty acid methyl esters significantly increased in all samples after digestion. These results demonstrated that relevant structural modifications may occur to the compounds found in frying oils during gastric digestion. Further investigation is crucial to assess the potential health implications of the compounds formed.

## 1. Introduction

Frying is one of the most used procedures for the preparation of foods. It is well-known that, during the frying process, a wide variety of chemical reactions occurs in the oil, globally referred to as thermoxidation, which results in the formation of a myriad of new compounds. Mechanisms of thermoxidation and analysis of the compounds formed in frying have been extensively reviewed [1,2,3,4,5] and are briefly outlined as follows. The initiation step of thermoxidative alteration consists of alkyl radical formation in the carbon adjacent to the double bond and the propagation step involves the addition of oxygen to form alkylperoxyl radicals and, hence, hydroperoxides. The high temperatures typical of frying lead to a decrease in oxygen pressure. Consequently, the concentration of alkyl radicals exceeds that of alkyl peroxyl radicals and decomposition of hydroperoxides becomes faster than formation. Breakdown, decomposition and further reactions of hydroperoxides and the radicals involved result in more stable non-volatile oxidation products and volatile compounds. From the nutritional point of view, the non-volatile compounds are essential since they form part of the lipid fraction of the fried product. From the major constituents of oils, i.e., triacylglycerols (TAG), virtually all the non-volatile compounds formed by thermoxidation show higher polarity than that of original TAG. That is the reason why determination of the polar compound fraction is the method recommended to evaluate the level of alteration in used frying oils, being the limit for human consumption established at 24–27% in countries with regulations [6,7]. Polar compounds can be separated according to molecular size into oxidized TAG monomers (oxTAGM) and TAG polymers. OxTAGM include a multitude of TAG which have in common that they contain one or more extra oxygenated function such as hydroperoxy, hydroxy, keto, epoxy, aldehyde, n-oxo, etc. TAG polymers form through interaction of two or more TAG radicals and include a large number of specific structures. TAG polymers can be further differentiated into TAG dimers (TAGD) and TAG higher oligomers (TAGO), constituted by two or more TAG molecules, respectively. Overall, TAG polymers account for a major fraction of the alteration compounds found, reaching as much as 12% on total oil at the limit of rejection of used frying oils.

Consumption of fried products has raised increasing concern regarding potential health issues, especially derived from the alteration of the oil absorbed, as reviewed in recent years [8,9,10,11,12,13]. Studies on the fraction of polar compounds of oils used in frying carried out in cell cultures showed reduction of HepG2 cell survival and disorder of lipid metabolism [14]. In mice, high amounts of polar compounds in diets impaired glucose tolerance and changed lipid deposition in liver and adipose tissues [15], and led to altered serum and hepatic metabolites [16]. Regarding the groups of compounds included in the polar compound fraction, it is known that oxTAGM shows high pancreatic hydrolysis rate and absorption, similar to those found for intact TAG [17,18,19,20,21]. In an interesting study, Li et al. isolated the groups of oxTAGM, TAGD and TAGO of the polar fraction and showed that oxTAGM can suppress proliferation of human hepatocellular carcinoma HepG2 and induce cell apoptosis. To a lesser extent, TGD and TAGO had the same effects [22]. Regarding TAGD and TAGO, lower absorption and digestibility values as compared with those of intact TAG and oxTAGM have been reported [17,18,19,20]. Nevertheless, the values obtained were higher than those expected considering the steric hindrance that pancreatic lipase finds to attach and hydrolyze them [21]. Thus, true digestibility values ranging from 23 to 49% and from 24 to 37% were found for oxidized fatty acid dimers and higher oligomers, respectively [17]. Even higher values were reported more recently for TAGD and TAGO in short-time digestibility assays [20]. A possible explanation for these observations could be that depolymerization reactions occur during their transit through the gastrointestinal tract.

In terms of the specific oxygenated functions formed in the fatty acyls of thermoxidized TAG, cytotoxic effects of monoepoxy linoleate or leukotoxin and its corresponding diol, leukotoxindiol, have been reported [23]. More recent studies have shown that epoxy stearic acid affected lipid accumulation and metabolism in HepG2 cells [24] and that a model TAG containing an epoxy function enhanced intestinal permeability in dextran sulfate sodium-induced colitis mice [25]. A myriad of deleterious effects have been attributed to hydroperoxides but, as commented in the Introduction, they decompose rapidly at the high temperatures of frying. Volatile, short-chain aldehydes, especially α,β-unsaturated aldehydes such as 4-hydroxy-2-nonenal, are nowadays of the greatest toxicological interest for their high reactivity with critical proteins and DNA leading to adducts formation [26,27,28,29].

Once ingested and prior to absorption, the chemical changes occurring to the multitude of compounds formed during frying have not been yet reported. Such changes would be essential to know because they may affect their nutritional properties or lead to formation of other compounds with possible adverse effects. In this context, it is well-known that dietary lipids may oxidize in the stomach, as evidenced in many studies collected in reviews published in recent years [30,31,32]. However, fewer studies, discussed as follows, have been published on the structure modifications undergone by oxidized lipids under gastrointestinal conditions and neither of them used oils subjected to the high temperatures of frying. Using triolein hydroperoxides, Kanazawa and coworkers were the first to report the conversion of hydroperoxides into aldehydes, alcohols, epoxyketones and others in experiments carried out in rats [33,34]. Later, similar results were obtained for oxidized oils subjected to in vitro gastrointestinal conditions, likewise reporting that some of the hydroperoxides originally present in oxidized oil were reduced to hydroxyl derivatives, epoxides and aldehydes [35,36,37]. Our research group further concluded that the quantitatively relevant structural changes of hydroperoxides were exclusively attributable to the very low pH characteristic of the stomach [38]. This work also showed that methyl 9,10-epoxystearate was transformed into the corresponding diol under simulated gastric conditions while its keto and hydroxy counterparts remained unaltered. Recently, the catabolic rates of labeled hydroperoxides were determined by measuring the expired ^13^CO^2^ levels and support that they decompose under gastric conditions to form a variety of degradation products including medium-chain compounds [39].

With all this in mind, the objective of the present work was to investigate for the first time the oxidation and depolymerization events which may occur to frying oils under simulated gastric conditions. Samples of sunflower oils used in frying with different alteration levels were selected and subjected to in vitro digestion using a classical in vitro model. Through combination of adsorption chromatography (silica minicolumns) and high-performance size-exclusion chromatography (HPSEC), changes in oxTAGM, TAGD and TAGO were evaluated. In parallel, derivatization to fatty acid methyl esters (FAME) and application of adsorption chromatography (solid-phase extraction) followed by gas–liquid chromatography with flame ionization detection (GC-FID) allowed quantitation of fatty acyls with specific oxygenated functions, i.e., epoxy, keto and hydroxy FAME.

## 2. Materials and Methods

### 2.1. Chemicals

Standards of 5-α-Cholestan-3-ol, and tocopherols (α, β, γ, δ) with a purity of 99% were provided by Sigma-Aldrich SA (St. Louis, MO, USA). Methyl heneicosanoate (C21:0) and methyl 12-hydroxystearate were supplied by Nu-Check-Prep (Elysian, MN, USA). Methyl trans-9,10-epoxystearate and methyl 12-oxostearate were purchased from Sigma–Aldrich (Steinheim, Germany). Silica gel 60 for column chromatography (particle size = 0.063–0.200 mm), sodium sulphate and platinum (IV) oxide hydrate were acquired from Merck (Darmstadt, Germany). All other chemicals and reagents (analytical grade) were purchased from local suppliers.

### 2.2. Samples

Refined sunflower oil and potatoes of the Agria variety were purchased locally. Potatoes were peeled, cut into homogeneous sticks (1 cm × 1 cm × 6 cm) and washed with water. Three domestic deep fryers (Moulinex AF2200, Lyon, France), each with a 1 L oil capacity, were used to fry 12 batches of 100 g potatoes at 175 ± 3 °C during 10 min with 20 min intervals between frying sessions. Therefore, 12 frying operations were carried out under conditions simulating discontinuous frying, following an established protocol [40]. Discontinuous frying is the deep-frying procedure used in domestic frying, restaurants and frying outlets. The experiments were conducted in triplicate without adding fresh oil throughout the process. Samples of five grams of frying oil were taken after each frying operation and stored in a nitrogen atmosphere at −18 °C until they were analyzed.

### 2.3. Digestion Assays

One hundred mL of oil emulsions (20 mg oil/mL) were prepared in a simulated gastric fluid (SGF) formulated as described in the United States Pharmacopeia [41]. The composition of SGF was 3.2% *w*/*v* pepsin in 0.03 M NaCl and 0.1 M HCl (pH 1.2). Emulsions adjusted to pH 6.6, the lowest pH considered neutral, were also prepared. Homogenization was performed in an Omnimixer (Sorvall, Newton, PA, USA) at 10,000 rpm for 5 min at 1 min intervals, as described elsewhere [42]. Gastric digestion was simulated using a classical in vitro model [38]. Triplicate emulsions were incubated in a shaking water bath for 3 h at 37 °C/100 rpm.

### 2.4. Lipid Extraction

Lipids were thoroughly extracted with 1:1 hexane diethyl ether after vigorous shaking in a vortex and centrifugation at 2000 rpm [42]. The extraction procedure was repeated three times, and the organic extracts were pooled and washed with distilled water. After filtration through anhydrous sodium sulfate, the solvent was evaporated in a rotatory evaporator under reduced pressure at room temperature and the extracted lipids were dried using a stream of nitrogen. The extraction yield ranged from 92 to 96%.

### 2.5. Analytical Determinations

#### 2.5.1. Acidity

The acidity was determined in the initial sunflower oil using titration according to ISO 660:2020 method [43] and expressed as percentage of oleic acid.

#### 2.5.2. Peroxide Value

The peroxide value was determined in the initial sunflower oil, used frying oils and oils extracted after gastric digestion, using the iodometric assay following ISO 3960:2017 method [43] and expressed as milliequivalents of oxygen per kg of oil.

#### 2.5.3. Oil Stability Index

The oil stability index (hours) was determined in the initial sunflower oil using a Rancimat device (Rancimat 743 equipment, Metrohm, Herisau, Switzerland) at 110 °C following AOCS Official Method Cd-12b-92 [44].

#### 2.5.4. Smoke Point

The smoke point temperature (°C) was measured in the initial sunflower oil according to AOCS Official Method Cc-9a-48 [44].

#### 2.5.5. Fatty Acid Composition

The fatty acid composition (%) was determined in the initial sunflower oil using gas chromatography after derivatization to fatty acid methyl esters using 2M KOH in methanol, following IUPAC Standard Methods 2.301 and 2.302 [45].

#### 2.5.6. Sterols

Individual and total sterol contents (mg/kg oil) were analyzed in the initial sunflower oil using gas chromatography following ISO 12228-1:2014 method [43] and 5-α-cholestan-3-ol was used as internal standard.

#### 2.5.7. Tocopherols

Tocopherols (mg/kg oil) were analyzed in the initial sunflower oil using high-performance liquid chromatography with fluorescence detection according to IUPAC Standard Method 2.432 [45]. Tocopherol standards were used for external calibration.

#### 2.5.8. Polar Compounds

Total content and distribution in different groups of polar compounds (weight % on oil) were determined in the initial sunflower oil, used frying oils and oils extracted after gastric digestion using silica column chromatography followed by analysis by HPSEC-RID according to Dobarganes et al. [46].

**Figure 1:**
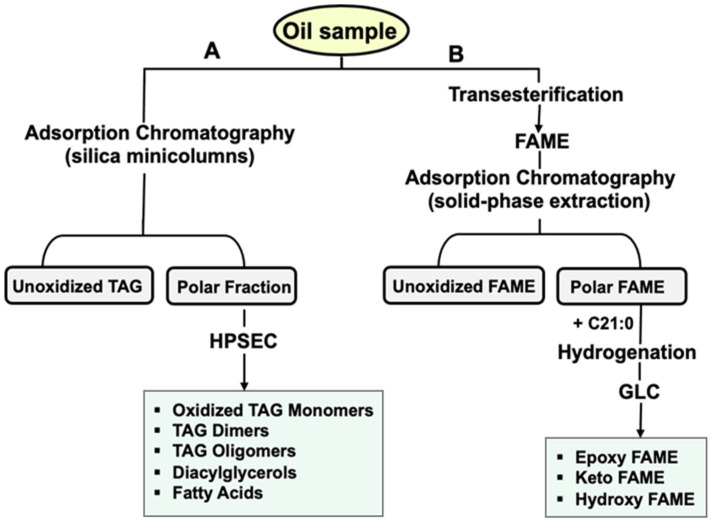
Analytical schemes of the methodologies A (described in Section 2.5.8 Polar Compounds) and B (described in Section 2.5.9 Epoxides, Ketones and Hydroxides).

Briefly, polar compounds were separated from the non-oxidized triacylglycerols using silica minicolumns. Five hundred milligrams of oil were dissolved in 5 mL of hexane and the non-oxidized triacylglycerols were eluted with 60 mL hexane/diethyl ether (90:10, *v*/*v*). After that, the polar fraction was eluted with 50 mL diethyl ether. The solvent of the polar fraction was evaporated in a rotary evaporator and redissolved in 1 mL diethyl ether. The polar fraction was analyzed by HPSEC in a liquid chromatograph equipped with a Rheodyne injector with a 20 µL sample loop, a Waters 510 pump (Waters, Milford, MA, USA), and a Waters 2414 refractive index detector. The separation was performed on two PLgel columns (Agilent Technologies, Palo Alto, CA, USA) packed with 5 µm particles of 100 and 500 Å pore size, respectively, and placed into an oven set at 35 °C. HPSEC-grade tetrahydrofuran was the mobile phase with a flow of 1 mL/min (isocratic elution mode). Resolved peaks of TAGO, TAGD, oxTAGM, diacylglycerols (DAG), and a peak corresponding to free fatty acids (FFA) and polar unsaponifiable matter were obtained. Total polar compounds were calculated as the sum of all the groups of compounds quantitated. Figure 1 shows a schematic representation of the analytical methodology used (A).

#### 2.5.9. Epoxides, Ketones and Hydroxides

FAME were obtained in the initial sunflower oil, used frying oils and oils extracted after gastric digestion by transmethylation with sodium methoxide at room temperature according to Berdeaux et al. [47]. Then, FAME were fractionated by solid-phase extraction using a silica cartridge (1 g) (Sep-Pak cartridges, Waters, Milford, MA, USA) into a fraction containing the non-oxidized FAME and a polar fraction, and further analyzed by GLC-FID. This methodology was developed and published by Marmesat et al. [48].

Briefly, 100 mg of FAME was dissolved in 2 mL of hexane. The fraction containing the non-oxidized FAME was eluted with 15 mL hexane/diethyl ether (98:2, *v*/*v*). The polar fraction was eluted with 25 mL of diethyl ether and then the solvent was evaporated under nitrogen. The polar fraction was dissolved in 2 mL of methanol and hydrogenated using platinum (IV) oxide hydrate as a metal catalyst and bubbling hydrogen at room temperature for 10 min. Then, methanol was evaporated, and a volume of 1 mL of a *tert*-butyl methyl ether solution containing 500 µg/mL of C21:0, used as internal standard, was added.

Analysis of epoxides, ketones and hydroxides was carried out by GC-FID using an HP 6890 Series chromatograph (Hewlett-Packard, Avondale, PA, USA) equipped with a split-splitless injector operating in the split mode with a 40:1 split ratio at 250 °C, a J&W DB-Wax fused-silica capillary column, 60 m × 0.25 mm I.D., film thickness 0.25 μm (J&W Scientific, Folsom, CA, USA) and a flame ionization detector at 250 °C. The analyses were run using hydrogen as carrier gas at 1 mL min^−1^ and under isothermal conditions using 230 °C for 25 min. Methyl *trans*-9,10-epoxystearate, methyl 12-oxostearate and methyl 12-hydroxystearate were used to obtain the response factors applied for quantification of epoxy FAME, keto FAME and hydroxy FAME relative to the IS (C21:0), expressed as mg/g FAME. Figure 1 shows a schematic representation of the analytical methodology used (B).

### 2.6. Statistical Analysis

Initial samples were analyzed in triplicate. All the experiments were performed in triplicate and the data were expressed as means ± standard deviations. One-factor ANOVA was applied using the 24.0 SPSS Statistics program (SPSS Inc., Chicago, IL, USA). Tukey’s test was used for comparisons between means and significance was defined at *p* < 0.05.

## 3. Results and Discussion

### 3.1. Characterization of Sunflower Oil

Table 1 shows characterization and quality parameters of the initial sunflower oil used for frying experiments. Sunflower oil ranks the fourth position in worldwide production, and it is the oil most used in Europe [49]. It was selected for this study as representative of an unsaturated oil used for frying prone to thermoxidation due to its high content in linoleic acid. The fatty composition, tocopherols and sterols contents of the oil were within the range normally found [49]. Regarding quality parameters, namely, acidity, peroxide value, oxidative stability index and smoke point, values were within those characteristic of good quality refined oils.

Table 2 shows polar compounds content and distribution of the initial sunflower oil and frying sunflower oil samples selected for digestion experiments, which corresponded to the second (SO1), fourth (SO2), sixth (SO3), ninth (SO4) and twelfth (SO5) frying operations.

In most countries with regulations for frying fats and oils, the level of 24 to 27% polar compounds is the limit established for human consumption [7]. The selection of frying oil samples in this study was intended to include oils with increasing amounts of polar compounds up to the limit of rejection (sample SO4), and beyond that (sample SO5). Total polar compounds, oxTAGM, TAGD and TAGO increased significantly in all samples. Polymerization started from the beginning of the frying process, and after the fourth frying operation (Frying SO2) TAGD together with TAGO made up for most of the polar compounds formed. Hydrolysis products, namely, DAG and FFA, remained practically at the same levels. It is well known that such compounds do not normally show significant increases during frying.

### 3.2. Changes in Peroxide Values, oxTAGM, TAGD and TAGO After Digestion

**Figure 2:**
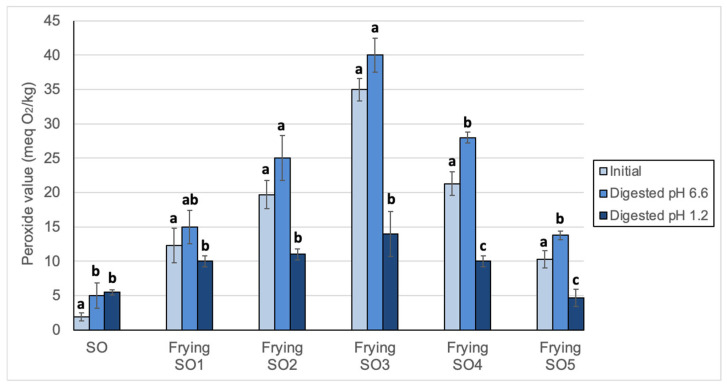
Peroxide values of sunflower oil (SO) and used frying sunflower oils (Frying SO1, SO2, SO3, SO4 and SO5) before and after in vitro digestion at pH 6.6 and 1.2. Results are expressed as Means ± SD (*n* = 3). Different letters between each initial and digested oils indicate significant differences according to Tukey’s test (*p* < 0.05).

**Figure 3:**
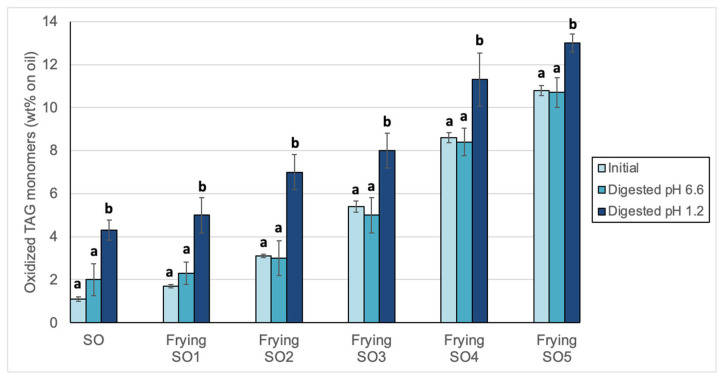
Oxidized triacylglycerol monomers (oxTAGM) in sunflower oil (SO) and used frying sunflower oils (Frying SO1, SO2, SO3, SO4 and SO5) before and after in vitro digestion at pH 6.6 and 1.2. Results are expressed as Means ± SD (*n* = 3). Different letters each initial and digested oils indicate significant differences according to Tukey’s test (*p* < 0.05).

**Figure 4:**
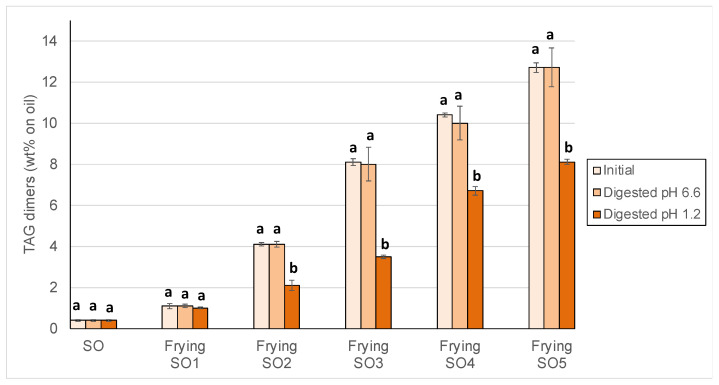
Triacylglycerol dimers (TAGD) in sunflower oil (SO) and used frying sunflower oils (Frying SO1, SO2, SO3, SO4 and SO5) before and after in vitro digestion at pH 6.6 and 1.2. Results are expressed as Means ± SD (*n* = 3). Different letters between each initial and digested oils indicate significant differences according to Tukey’s test (*p* < 0.05).

**Figure 5:**
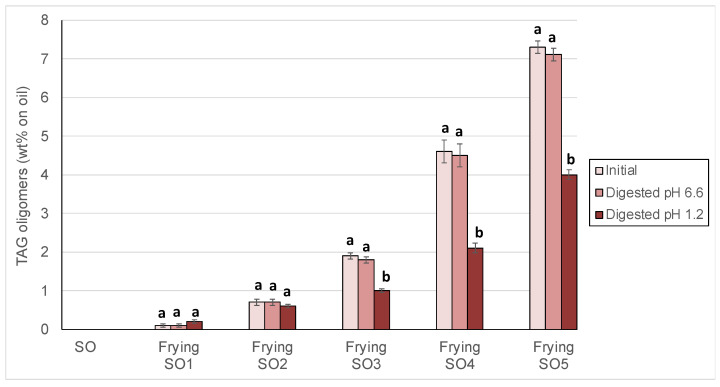
Triacylglycerol higher oligomers (TAGO) in sunflower oil (SO) and used frying sunflower oils (Frying SO1, SO2, SO3, SO4 and SO5) before and after in vitro digestion at pH 6.6 and 1.2. Results are expressed as Means ± SD (*n* = 3). Different letters between each oil and samples digested indicate significant differences according to Tukey’s test (*p* < 0.05).

Figure 2, Figure 3, Figure 4 and Figure 5 show data of peroxide values, oxTAGM, TAGD and TAGO of initial and used frying sunflower oil samples before and after in vitro digestion, at both pH 1.2 and 6.6. A value of pH 1.2 is considered ultra-acidic and it is described in the formulation of simulated gastric fluid, while pH 6.6 is the more acidic value in the range of neutral pH (see Section 2.3 in Section 2 Materials and Methods). Considerable pH variations may occur under human digestion. Ingestion of food with buffering capacity and basic liquids like milk can increase the pH up to 7, which then time dependently declines to acidic values [50]. Nevertheless, the procedure recommended for gastric pH adjustment in bioavailability studies is pH of 1–2 [51].

Peroxide value, an analytical index for measurement of hydroperoxides, is not recommended for analysis of used frying oils because hydroperoxides are unstable and intermediate compounds are not indicative of the alteration level. Thus, Figure 2 shows that values for initial oils increased after frying up to a certain level of alteration (SO3) and then decreased substantially. However, since potential oxidation under gastric conditions may occur at low temperature, peroxide value can provide essential information on the initial stage of oxidation. Results showed low but significant increases in the initial, unoxidized SO at both pHs. For used frying oils digested at pH 6.6, peroxide values increased in all samples, although only significantly in SO4 and SO5. Quite in contrast, all frying samples showed significant decreases at pH 1.2. It is not easy to explain the results obtained because hydroperoxides may be forming and decomposing under the digestion conditions used. Still, these results indicated that formation surpassed decomposition in the unoxidized SO while the opposite occurred to frying oils, but only at the ultra-acidic pH characteristic of the gastric environment.

As commented in the Introduction, many studies have been published in recent years showing that oils can oxidize under gastric conditions. Our results on unoxidized SO agreed with those reporting an increase in hydroperoxides after digestion of unoxidized oils [52,53,54]. Also, when testing oxidized lipids, decreases of hydroperoxide levels were normally found either in vivo [33,34,55] or in vitro [37,56]. In our last publication on this subject, minimum losses of hydroperoxides of between 54 and 62% were found after simulated gastric digestion of methyl linoleate hydroperoxides at pH 1.2. Considering that the pH of human gastric fluid ranges from 1 to 3.5, samples were tested at pH 3.0 and, likewise, high minimum losses were found, ranging from 42 to 58% [38].

When hydroperoxides were found in the atherogenic plaque, considerable attention was paid to the absorption of hydroperoxides in vivo, speculating that oxidized lipids in the diet could be one of the sources. However, the studies discussed above give evidence that most dietary hydroperoxides are decomposed or converted to other compounds during gastric digestion. In an interesting article recently published, Takahashi and coworkers performed a lymph duct-cannulation study in rats using deuterium-labeled trioleoyl glycerol hydroperoxides. After administration, only unlabeled TAG hydroperoxides were detected in the lymph, indicating that dietary hydroperoxides decomposed rapidly in the gastrointestinal environment and the hydroperoxides found in the lymph were produced in vivo [57].

Following gastric digestion, decomposition or further reaction of hydroperoxides to yield secondary oxidation compounds, such as aldehydes, epoxides, ketones and hydroxides, have been reported [33,34,37,38,56].

In the present study, evaluation of oxTAGM was carried out to provide a complete measurement of the TAG bearing both hydroperoxyl functions and oxygenated functions characteristic of secondary oxidation in at least one of the fatty acyl groups of the molecule [58]. Such secondary oxygenated functions are mainly epoxy, keto, hydroxy and n-oxo. OxTAGM would also include polyoxygenated compounds such as epoxyhydroxy, dihydroxy, trihydroxy and others reported to be found [59]. Results obtained showed the significant increase of oxTAGM in all samples, including the initial, unoxidized SO, after digestion at pH 1.2. (Figure 3). These results support that oxidation events may occur to both unoxidized and used frying oils under gastric digestion due to the acidic conditions.

Besides oxTAGM, volatile compounds may be formed from the β-scission of the alkoxyl radicals coming from hydroperoxides. In fact, short-chain aldehydes, alkenes, alkanes and other volatiles have been detected after in vitro gastric digestion [38,39,60,61,62,63].

Changes of polymeric TAG, separately quantitated as TAGD and TAGO, are shown in Figure 4 and Figure 5.

**Figure 6:**
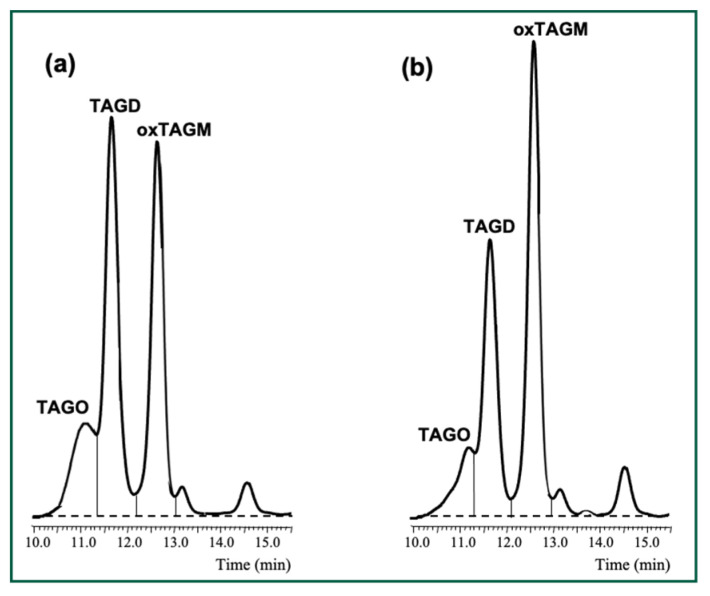
High-performance size-exclusion chromatograms of polar fractions of a sample of used frying sunflower oil SO4 before (**a**) and after (**b**) in vitro gastric digestion at pH 1.2. Abbreviations: TAGO, triacylglycerol oligomers; TAGD, triacylglycerol dimers; oxTAGM, oxidized triacylglycerol monomers.

Results showed that TAGD (Figure 4) and TAGO (Figure 5) significantly decreased in most samples at pH 1.2, which was especially notable in SO3, SO4 and SO5. TAGD contents in such samples were reduced by between 30 and 50% and even higher losses of between 50 and 70% were found for TAGO. To the best of our knowledge, this is first report on depolymerization events occurring under in vitro gastric conditions in high-molecular weight compounds formed during frying. For illustration, Figure 6 shows the chromatogram of the polar fractions of one sample of used frying SO4 before (a) and after (b) digestion. As can be clearly observed, the significant decrease of the peaks of TAGO and TAGD was concomitant with the increase of the peak of oxTAGM. Efforts made to identify some of the high molecular-weight structures formed at frying temperatures have revealed the presence of dimers, trimers, and tetramers containing one, two, or three additional oxygens, also with and without additional sites of unsaturation [64]. The analytical methodology used in the present study allows quantitation of dimers and higher oligomers but does not permit to quantitate trimers and tetramers separately.

The combined results obtained for oxTAGM, TAGD and TAGO indicate that the considerable increase of oxTAGM after digestion can be attributed in great part to the breakage of TAGD and TGAO. Also, a fraction of TAGD is anticipated to originate from TAGO.

The main types of bonds between TAG reported to be found in dimers and higher oligomers produced by oxidation and thermoxidation are peroxy-linked, ether-linked and carbon-linked [6,65,66]. Other bonds, i.e., epidioxides and cyclic bonds [50] and ester bonds [67,68] have been also proposed as linkages in polymers of oil and fatty acid esters. Peroxy-linked dimers and higher oligomers are mainly formed at low or moderate temperatures and are rather unstable at elevated temperatures (above 100 °C) producing either ether-linked or other oxygenated polar dimers [6,69]. Since carbon linkages are very stable, breakage of ether and/or other oxygenated linkages may account for the depolymerization effects observed in the present study. The reductive cleavage of the ether linkage of TAG dimers under acidic conditions was already reported in the 1970s [70,71]. Specifically using hydrochloric acid, TAG dimers rendered TAG monomers with a hydroxy function [71]. However, the extent of this reaction is unknown under simulated gastric conditions and other linkages may be also responsible for the depolymerization observed. It is clear that much research remains to be done to determine the quantitative relevance of the different types of linkages reported to be formed between TAG during frying and the mechanisms involved in their cleavage under acidic conditions.

As commented in the Introduction, the possible breakage of dimers and higher oligomers could explain the unforeseen high digestibility values found for such compounds despite their low hydrolysis rate obtained after pancreatic lipolysis [21]. Values of true digestibility as high as 49% and 37% were first unexpectedly found for oxidized fatty acid dimers and higher oligomers, respectively, in long-term in vivo studies [17]. In another study, thermoxidized radiolabeled linoleic acid was included in diets fed to rats and results confirmed high digestibility values for dimers and oligomers. Furthermore, nonaltered radiolabeled fatty acids, which were absent in diets, were found in feces, thus indicating that depolymerization reactions in the stomach could have taken place [18]. Such high digestibility values were later confirmed in short-term in vivo studies using esophageal probes, showing values of about 50% [19]. Even higher values, as high as 81 and 58%, were reported years later for TAG dimers and higher oligomers in short-time digestibility assays [20].

### 3.3. Changes in Unoxidized TAG After Digestion

**Figure 7:**
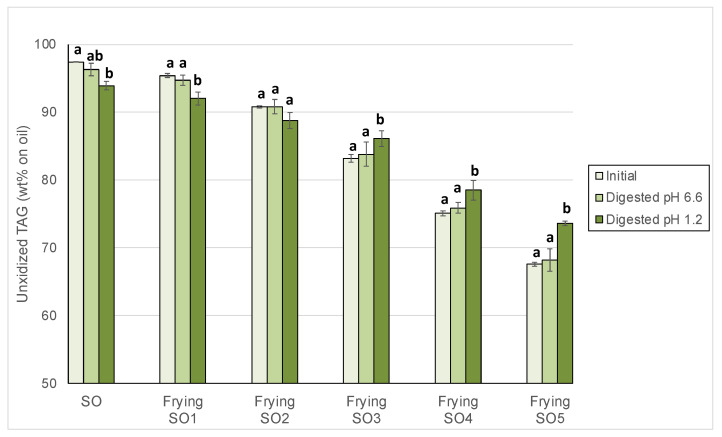
Unoxidized triacylglycerols (TAG) in sunflower oil (SO) and used frying sunflower oils (Frying SO1, SO2, SO3, SO4 and SO5) before and after in vitro digestion at pH 6.6 and 1.2. Results are expressed as Means ± SD (*n* = 3). Different letters between each initial and digested oils indicate significant differences according to Tukey’s test (*p* < 0.05).

Results obtained for changes of unoxidized TAG after gastric digestion showed significant decreases in SO and SO1, albeit significant increases in those used frying oils which underwent the largest extent of depolymerization, i.e., SO3, SO4 and SO5 (Figure 7). These findings add evidence to support the occurrence of depolymerization since the only possible explanation for these results is the release of unoxidized TAG from the breakage of TAGD and TAGO.

### 3.4. Evaluation of Epoxy, Keto and Hydroxyl Fatty Acyls

Initial oils and oils digested at pH 1.2 were also analyzed to quantitate epoxy, keto and hydroxy FAMEs, originally as fatty acyls located in the TAG molecule (Table 3). It is important to note that this analysis allowed quantitation of mono-oxygenated FAMEs, which accounts for a relevant group in used frying oils [48,72,73].

Results obtained in the present study showed that levels of epoxy, keto and hydroxy FAMEs in oils before digestion were consistent with those reported for used frying sunflower oils [48,72,73,74,75,76]. Thus, even though epoxides are reactive, their amounts have been found to increase during the frying process, as reported using either model triacylglycerols or oils [72,74,75,76].

The main route of formation of epoxides at frying temperatures is from hydroperoxides reacting at the site of double bonds. This was proposed after finding that only the compounds formed when the oxygen added across an existing double bond were detected by GLC-MS at 180 °C. Thus, two saturated epoxides, *trans*-9,10- and *cis*-9,10-epoxystearate, were formed in methyl oleate and triolein samples and four monounsaturated epoxides, *trans*-12,13-, *trans* 9,10-, *cis*-12,13-, and *cis*-9,10-epoxyoleate, were formed in methyl linoleate and trilinolein samples [73,74].

After digestion of the initial, unoxidized SO, epoxy and hydroxy FAMEs were formed. Regarding used frying SO samples, only hydroxy FAMEs showed significant increases in all samples, even though TAG containing epoxy, keto and hydroxy functions could all have formed from further oxidation of hydroperoxides produced under gastric conditions. This could be due in part due to the additional expected increase in TAG bearing the hydroxy group after digestion, deriving from breakage of TAGD and TAGO linked by oxygenated bonds. In the case of epoxy compounds, the opening of the oxirane ring to yield diols may have contributed to finding lesser amounts than expected after digestion. In this context, a study carried out using model epoxy methyl linoleate revealed that at least 50% was lost after in vitro gastric digestion at pH 1.2 and concomitant formation of dihydroxy methyl linoleate could be detected [38]. The same study showed that keto and hydroxy methyl linoleate remained unaltered after digestion.

The research works closest to the present study have shown the generation of hydroxy-octadecadienoates during in vitro digestion of slightly oxidized sunflower oil, first reported by Nieva-Echevarria et al. [35]. The most recent article published by this research group was carried out using highly oxidized soybean oil and showed that the hydroperoxide level declined throughout the process of digestion while epoxy and hydroxy-compounds, keto-dienes, furan-derivatives and n-alkanals persisted or even increased [37]. The results obtained in the present study are consistent with those published in such research works, although other findings are reported here for used frying oils, in which dimers and higher oligomers are abundant, showing a significant extent of depolymerization.

## 4. Conclusions

To the best of our knowledge, this work shows for the first time that structural modifications occur in compounds formed during frying under simulated gastric conditions. Samples of used frying sunflower oil obtained in successive frying operations with a significant increase in polar compounds were tested. Results showed that significant depolymerization events occurred under in vitro gastric digestion conditions, even in samples below the limit of polar compounds for human consumption established in regulations. Although oxidation events were also observed, especially in the unoxidized oil, depolymerization prevailed in most used frying oils giving rise to a significant decrease in TAGO (50–70%) and TAGD (30–50%), in parallel to the increase in unoxidized TAG. In terms of specific oxygenated functions, the marked increase found in hydroxy FAMEs after digestion (38–61%) stood out. This finding could be attributed in great part to the breakage of oxygenated bonds between TAG molecules of TAGO and TAGD. Clearly, results showed that the acidic gastric environment is the key factor in the chemical changes found. Altogether, the results obtained provided a foothold to better understand the complex reactions occurring under gastric conditions. Much research is needed to unravel the modifications of frying oils throughout the gastrointestinal tract before absorption into the bloodstream, which would have a great impact on their nutritional quality and health effects. In this context, further studies are underway in our laboratory to identify and quantify specific compounds resulting from depolymerization in the gastric environment and to reveal the mechanism of cleavage of bonds between TAG forming dimers and higher oligomers.

## Figures and Tables

**Table 1:** Characterization and quality parameters of the initial sunflower oil.

Analytical Determination	Sunflower Oil
Fatty acid composition (%)	
C14:0	0.08 ± 0.00
C16:0	6.02 ± 0.00
C16:1	0.09 ± 0.00
C17:0	0.03 ± 0.00
C18:0	3.73 ± 0.01
C18:1	29.31 ± 0.01
C18:2	58.83 ± 0.01
*Trans* C18:2	0.10 ± 0.00
C18:3	0.06 ± 0.00
C20:0	0.22 ± 0.01
C20:1	0.14 ± 0.01
C20:4	0.01 ± 0.00
C22:0	0.57 ± 0.01
C24:0	0.11 ± 0.01
Others	0.70 ± 0.01
Tocopherols (mg/kg)	627 ± 14
Sterols (mg/kg)	3219 ± 25
Acidity (% oleic acid)	0.04 ± 0.01
Peroxide value (meq O_2_/kg)	5.7 ± 1.0
Oxidative stability index (h)	5.3 ± 0.3
Smoke point (°C)	230 ± 2

Results are expressed as Means ± SD (*n* = 3).

**Table 2:** Total content and distribution of polar compounds in initial and used frying sunflower oils.

Polar Compounds	Total (wt %)	oxTAGM (wt %)	TAGD (wt %)	TAGO (wt %)	DAG (wt %)	FFA (wt %)
Initial SO	2.7 ± 0.1 a	1.1 ± 0.1 a	0.4 ± 0.0 a	nd	0.8 ± 0.1 a	0.4 ± 0.0 a
Frying SO1	4.6 ± 0.3 b	1.7 ± 0.0 b	1.1 ± 0.0 b	0.1 ± 0.0 a	1.1 ± 0.1 b	0.5 ± 0.0 a
Frying SO2	9.4 ± 0.2 c	3.1 ± 0.4 c	4.2 ± 0.1 c	0.7 ± 0.1 b	1.0 ± 0.0 b	0.4 ± 0.0 a
Frying SO3	16.8 ± 0.3 d	5.6 ± 0.2 d	7.9 ± 0.2 d	1.8 ± 0.1 c	1.1 ± 0.3 b	0.4 ± 0.1 a
Frying SO4	24.7 ± 0.4 e	8.6 ± 0.2 e	10.5 ± 0.3 e	4.3 ± 0.1 d	0.8 ± 0.1 a	0.5 ± 0.1 a
Frying SO5	32.3 ± 0.5 f	10.9 ± 0.3 f	12.5 ± 0.2 f	7.3 ± 0.2 e	1.1 ± 0.2 b	0.5 ± 0.1 a

Abbreviations: oxTAGM, oxidized triacylglycerol monomers; TAGD, triacylglycerol dimers; TAGO, triacylglycerol oligomers; DAG, diacylglycerols; FFA, free fatty acids; SO, sunflower oil. Results are expressed as Means ± SD (*n* = 3). Frying SO1, SO2, SO3, SO4 and SO5 samples correspond to the second, fourth, sixth, ninth and twelfth discontinuous frying operations, respectively. Different letters in the same column indicate significant differences according to Tukey’s test (*p* < 0.05).

**Table 3:** Epoxy FAME, keto FAME and hydroxy FAME in initial and digested (pH 1.2) sunflower oils.

Oil	Epoxy FAME (mg/g Oil)	Keto FAME (mg/g Oil)	Hydroxy FAME (mg/g Oil)
Initial SO	nd	nd	nd
Digested SO	0.15 ± 0.06	nd	0.94 ± 0.19
Initial Frying SO1	0.83 ± 0.12 a	0.24 ± 0.04 a	0.80 ± 0.08 a
Digested Frying SO1	0.50 ± 0.22 a	0.46 ± 0.11 b	2.07 ± 0.33 b
Initial Frying SO2	1.58 ± 0.06 a	0.82 ± 0.06 a	1.95 ± 0.19 a
Digested Frying SO2	1.95 ± 0.07 a	1.53 ± 0.12 b	3.13 ± 0.37 b
Initial Frying SO3	2.93 ± 0.09 a	1.50 ± 0.16 a	3.17 ± 0.29 a
Digested Frying SO3	3.57 ± 0.29 a	1.85 ± 0.35 a	6.27 ± 0.38 b
Initial Frying SO4	4.53 ± 0.12 a	2.43 ± 0.17 a	5.53 ± 0.25 a
Digested Frying SO4	5.23 ± 0.56 a	2.77 ± 0.25 a	9.23 ± 0.56 b
Initial Frying SO5	5.67 ± 0.17 a	3.03 ± 0.21 a	6.03 ± 0.21 a
Digested Frying SO5	6.50 ± 0.50 a	3.50 ± 0.41 a	9.97 ± 0.56 b

Abbreviations: FAME, fatty acid methyl esters; SO, sunflower oil. Different letters between each initial and digested oils indicate significant differences according to Tukey’s test (*p* < 0.05).

## Data Availability

The original contributions presented in this study are included in the article. Further inquiries can be directed to the corresponding author.
